# Breaking the silence: barriers to maternal healthcare utilisation among women in South-South Nigeria

**DOI:** 10.3389/fgwh.2025.1623067

**Published:** 2025-09-22

**Authors:** M. S. Ekpenyong

**Affiliations:** School of Nursing and Public Health, Manchester Metropolitan University, Brooks Building, Manchester, United Kingdom

**Keywords:** maternal healthcare, barriers, women's experiences, South-South Nigeria, qualitative research

## Abstract

**Background:**

Despite awareness of the benefits of facility-based deliveries, many women in Sub-Saharan Africa (SSA) still deliver outside healthcare settings, often without skilled birth attendants. Access to maternal healthcare encompasses affordability, physical accessibility, and acceptability.

**Objective:**

This study aimed to explore the factors influencing maternal healthcare utilisation in South-South Nigeria, with a focus on identifying the “silences” surrounding women's access to care, and understanding the facilitators, barriers, and suggested improvements.

**Methods:**

An exploratory qualitative design was adopted to investigate women's perceptions of the hidden factors influencing maternal healthcare utilisation. Data were collected from women of reproductive age (20–49 years) in one of the tertiary health hospitals in Nigeria. Eight semi-structured interviews were performed and transcribed. Data were analysed thematically using the Silences Framework.

**Results:**

Three themes emerged: facilitators, barriers, and suggestions. Facilitators included women's recognition of skilled providers and the safety offered by emergency care. Despite dissatisfaction, many continued facility use due to trust in medical expertise. Barriers included negative staff attitudes, breaches of confidentiality, domestic violence, financial constraints, and systemic inefficiencies. Stigma further silenced discussion of sensitive issues, reinforcing under-utilisation. Some women turned to traditional birth attendants or private clinics, valuing compassion despite costs or risks. Suggestions centred on staff training in compassionate care, improved monitoring, better equipment, and stronger policies to uphold dignity and privacy.

**Conclusion:**

The study emphasises the importance of addressing both systemic issues and interpersonal dynamics to improve maternal healthcare services. Women balance the perceived necessity of skilled care with negative experiences in formal facilities. While competence drives use, poor attitudes and systemic gaps reduce trust. Tackling both structural and interpersonal barriers is critical. Respectful, culturally sensitive care and stronger accountability are essential. These findings offer practical guidance for reforming maternal healthcare in Nigeria.

## Introduction

Maternal and child health are critical indicators of a society's developmental status and the effectiveness of its healthcare delivery system. The World Health Organisation (WHO) identifies three critical factors underpinning maternal deaths globally, chief among them being the lack of access to and utilisation of essential obstetric services (1). WHO in 2017 further reports a negative correlation between maternal mortality rates and maternal healthcare utilisation, estimating that 295,000 women died during pregnancy or childbirth, with 94% of these deaths occurring in low-income countries, deaths that were largely preventable through timely emergency obstetric care (2).

According to the World Health Organisation, globally, an estimated 830 women die each day from preventable pregnancy and childbirth-related causes, with 99% of these deaths occurring in developing countries (3). Maternal and infant mortality are critical indicators of national development, and Nigeria's record remains deeply troubling. Although the Sustainable Development Goals commit to reducing maternal deaths by 2030, maternal mortality continues to rise across much of sub-Saharan Africa. In Nigeria alone, approximately 59,000 women die annually from pregnancy-related complications, a rate that makes Nigerian women nearly 500 times more likely to die in childbirth than their counterparts in high-income countries (4). With a maternal mortality ratio of 545 per 100,000 live births, Nigeria ranks second globally after India and remains the worst affected in Africa. Alarmingly, this translates to at least one maternal death in every 20 live births, reflecting systemic weaknesses in the health system (4). Beyond medical causes, structural factors such as poverty, gender inequality, limited education, and the imposition of hospital user fees exacerbate risks. Consequently, many women, particularly in rural communities, increasingly rely on faith healers and traditional birth attendants as alternative sources of care (5).

A study across six Sub-Saharan African (SSA) countries reinforced that inadequate access to pregnancy and delivery care significantly contributes to global maternal and neonatal mortality (6). Antenatal care, skilled facility-based deliveries, and postpartum services remain critical indicators for tracking maternal outcomes, especially mortality. Despite awareness of the benefits of facility-based deliveries, many women in SSA still deliver outside healthcare settings, often without skilled birth attendants. Access to maternal healthcare encompasses affordability, physical accessibility, and acceptability (7, 8). Socioeconomic and geographical barriers continue to hinder maternal healthcare access (9, 10), and despite efforts to expand healthcare services, significant gaps in emergency obstetric care persist across diverse settings (11).

In developing countries, maternal mortality is also influenced by broader societal factors, including gender inequality, harmful cultural practices, and entrenched poverty (12–14). Despite high maternal mortality and underutilisation of maternal healthcare in Nigeria, limited qualitative research explores women's lived experiences, barriers, and facilitators influencing their healthcare-seeking behaviours, particularly in the South-South region. Given these realities, this study aimed to explore the thoughts, beliefs and emotions of women regarding the hidden “silences” affecting the utilisation of maternal healthcare services in South-South Nigeria.

## Materials and methods

### Study setting and design

The study was conducted in one of the tertiary hospitals in South-South Nigeria. Tertiary facility was selected because they serve as referral centres with a higher volume of obstetric cases, providing insights into women's interactions with formal healthcare systems. An exploratory qualitative design was adopted to investigate women's perceptions of the hidden factors influencing maternal healthcare utilisation. This approach, aimed at deepening understanding rather than offering definitive solutions (15), provides a foundation for future interventions (16).

### Study participants and sampling

Eligible participants included patients with direct obstetric complications that has given birth in the last one year and/or pregnant, age between 20 and 49 years and must be attending the health facility for care at the time of this study. Participants for this study were approached in the antenatal and postnatal wards through the hospital liaison officer. Potential research participants were provided with an information sheet and either signed a written consent form or gave verbal consent if they agreed to participate. The facility offered a full range of maternity services, and women with post-delivery complications, which are not uncommon in this setting, received ongoing follow up care here up to 12 months after delivery. The inclusion of women who had given birth in the previous year is on the premise that their experiences would help to enrich data collected. They were included in this study because the researcher hopes that their experiences would help to enrich the data collected. The research participants were married, single, divorced, separated or widowed.

Sample size determination was linked with the concept of data saturation. Participants were purposively selected based on age (20–49), recent birth (within 12 months), and willingness to participate. Exclusion criteria included severe illness, woment not in their reproductive age, women who were not willing to participate in the study even when they met all the study criteria. Recruitment of research participants occurred face-to-face at antenatal/postnatal clinics. Thematic saturation was achieved after the 7th interview, with no new themes emerging in the 8th. All participants were consenting patients currently accessing maternal health services.

### Data collection

Data were gathered through semi-structured interviews, focusing on the “silences” surrounding maternal healthcare use. The semi-structured interview guide was developed based on existing literature and expert consultation, and pilot-tested with two participants. Interviews, lasting 45–60 min, were conducted in private clinic rooms. The researcher spent approximately two weeks in the clinic environment before data collection, building rapport and trust through informal interactions. The exploratory nature of the interviews allowed in-depth engagement with participants' lived experiences.

### Ethical considerations

Prior to participation, each woman received a detailed information sheet explaining the purpose, procedures, and voluntary nature of the study. Participants were reminded of their autonomy to withdraw at any stage without consequence, and questions were phrased in culturally appropriate, non-directive language to reduce coercion. Written consent was obtained from those willing to sign a consent form, while verbal consent was recorded for participants who preferred this approach, under institutional ethical approval. Given the sensitivity of issues such as domestic violence, measures were also taken to safeguard participants emotionally. Interviews were held in private consultation rooms, ensuring confidentiality. Women were informed that they could decline to answer distressing questions. A referral pathway to counselling and social support services within the hospital was established for any participant who experienced distress or disclosed trauma. Although no participant required referral, these provisions were considered essential to maintaining ethical integrity. A reflexive journal was also maintained by the researcher to critically reflect on positionality and potential bias.

### Data analysis

**Figure 1:**
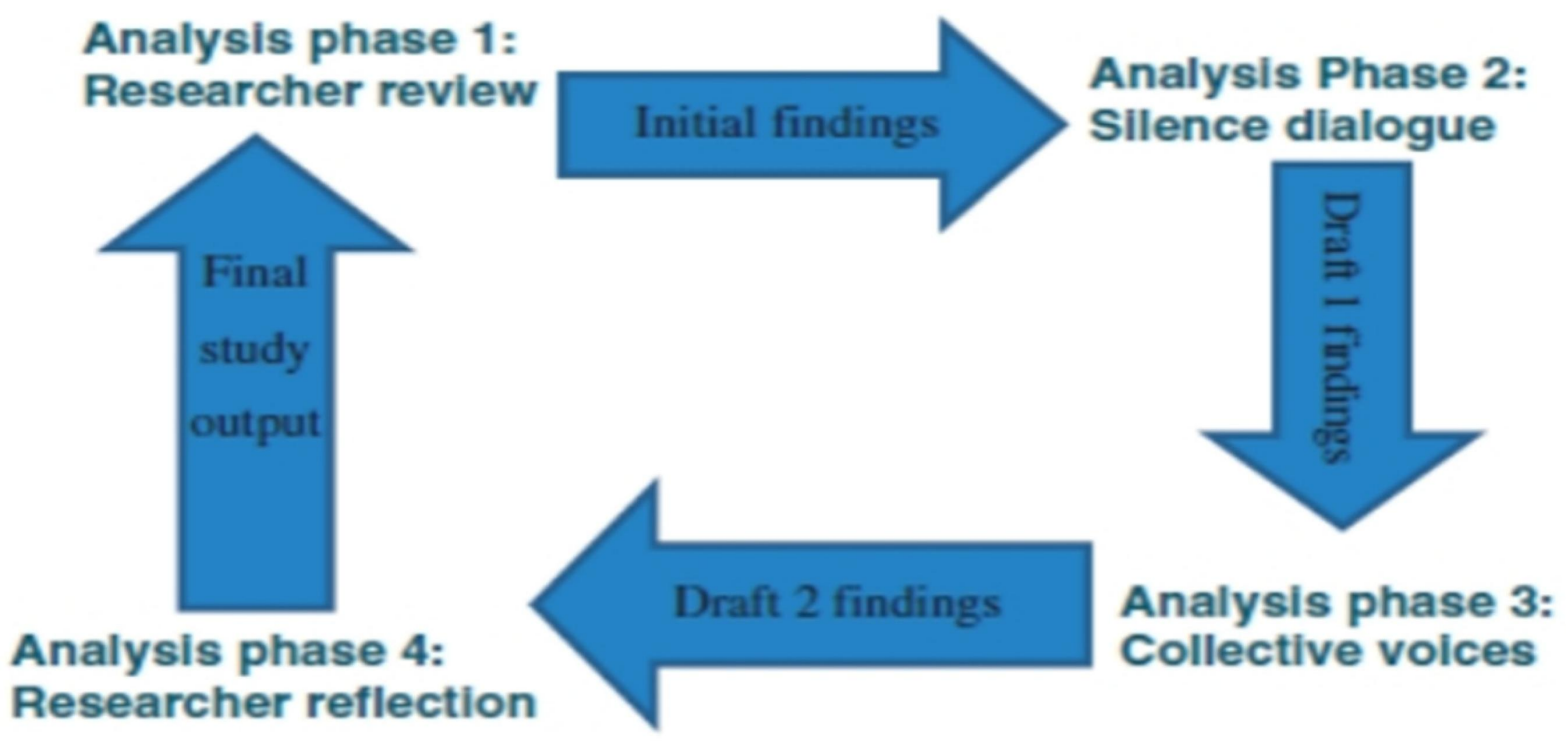
The silences framework. Adapted from Serrant-Green L. (17).

Interviews, conducted in English, were audio-recorded, transcribed verbatim, and analysed thematically using NVivo software. To ensure accuracy, transcripts were validated by participants. Pseudonym names were used to maintain confidentiality. The Silences Framework guided data analysis, progressing through four iterative phases involving participant validation, social network review, and researcher reflection (17) (Figure 1).

The Silences Framework seeks to acknowledge and redress the balance of power regarding what and whose experiences are considered in research (17). The Silences Framework was chosen because it uncovers unspoken, culturally embedded factors influencing maternal healthcare utilisation, capturing narratives often censored or normalised (18). It allows systematic exploration of barriers such as gendered power dynamics, confidentiality fears, and reproductive health stigma that traditional qualitative methods might miss. To deepen analysis, the study integrates structural violence and intersectionality. Structural violence theory highlights systemic social and institutional inequities constraining access to quality care. Intersectionality examines how overlapping identities, gender, socio-economic status, education, and location—shape women's experiences differently. Together, these lenses situate silenced narratives within broader social determinants and power hierarchies. This triangulated approach enhances interpretive validity, revealing both what is unspoken and why barriers persist. Ultimately, it enables nuanced, contextually grounded recommendations for improving maternal healthcare access.

In this context, this study aims to identify barriers to accessing maternal healthcare services from the lived experiences of the participants. The Silences Framework is considered appropriate for this research because it seeks to give voice to experiences, subjects, and issues that are often hidden, devalued, or 'silenced' in research, excluded from policy discourse, and marginalised in practice (17).

The four phases of the data analysis included:

**Phase 1:** Following transcription, the data from the interviews were analysed by the researchers, and recurrent themes were identified as the preliminary findings of the study.
**Phase 2:** The preliminary findings from Phase 1 were reviewed by the research participants in the presence of the researcher, who noted any comments, non-verbal expressions, and reflections. These were used to enhance further critique, confirming or refuting the findings from Phase 1. Member checking was conducted with five of the eight participants through individual follow-up meetings. Three local maternal health practitioners served as social-network reviewers. Feedback was incorporated when it aligned with the original themes, while discrepancies were deliberated in team meetings to achieve consensus.
A discussion of the findings was then formulated and carried forward to Phase 3 for further analysis.
**Phase 3:** Further analysis of the findings from Phase 2 was undertaken by the social networks of the research participants. The participants in this phase mirrored the original research participants and were drawn from one of the tertiary health facilities but had not taken part in the semi-structured interviews. The aim was to consolidate the findings from Phase 2 through a critically indirect, associative perspective. These participants were recruited at the same time as the original participants.
**Phase 4**: Finally, the researcher reflected on the findings from Phase 3, revisiting, reviewing, and developing emerging themes, which formed the final output of the study.

### Trustworthiness of the study

Trustworthiness was ensured through multiple strategies, including triangulation (interviews and member checking), an audit trail of coding decisions, peer debriefing, and a reflexive journal documenting researcher assumptions. Transcripts were returned verbatim to participants for member checking.

## Results

### Participant characteristics

Eight women participated in the study, ranging in age from 24 to 41 years, with parity from one to four children. Educational backgrounds varied from primary to tertiary level, reflecting a diversity of perspectives. Table 1 summarises participants' demographic characteristics.

**Table 1:** Participants characteristics.

Names of research participants	Age	Educational qualification	Marital status	Parity
Justina	24 years	Secondary education	Married	1
Chioma	32 years	University-Educated	Married	2
Mary	37 years	University-educated	Married	4
Amaka	29 years	University student	Single	1
Ada	34 years	Secondary education	Married	3
Ijeoma	41 years	Secondary education	Married	3
Ifeoma	30 years	University-educated	Separated	2
Udoka	36 years	Secondary education	Married	4

The following table summarises the major themes and their corresponding subthemes (minor themes) that emerged from the analysis of maternal healthcare utilisation among women in Nigeria (Table 2). These themes reflect both facilitators and barriers influencing women's decisions and experiences.

**Table 2:** Major and minor themes on maternal healthcare utilisation.

Major themes	Minor themes (Subthemes)
Facilitators of Maternal Healthcare Utilisation	- Recognition of skilled care as life-saving - Perceived competence of healthcare professionals - Trust in ability to handle complications and emergencies
Barriers to Maternal Healthcare Utilisation	- Negative attitudes and disrespect from healthcare workers - Mistreatment and neglect - Breaches of confidentiality - Stigma around reproductive health issues - Domestic violence and lack of empathetic response - Systemic inefficiencies (understaffing, long waiting times, inadequate equipment)
Alternative Care-Seeking Practices	- Use of private clinics for compassionate, personalised care (despite higher costs) - Turning to Traditional Birth Attendants (TBAs) for empathy, accessibility, and cultural familiarity
Suggestions for Improvement	- Training healthcare providers in compassionate and respectful care - Government monitoring and enforcement of standards - Improved staffing and availability of equipment - Policies safeguarding dignity, privacy, and confidentiality

### Facilitators to healthcare utilisation

#### Skilled care as life-saving

Despite dissatisfaction with aspects of healthcare services, women repeatedly emphasised the indispensable role of skilled care in preventing maternal and neonatal deaths. For many, the presence of trained staff and emergency equipment was the deciding factor in continuing to utilise formal healthcare facilities.

Justina (aged 24, secondary education) stated:

Despite some bad experiences I had with the health facility, I would still prefer to attend antenatal care and deliver at a healthcare facility. They are better equipped to handle emergencies than traditional birth attendants.

#### Trust in professional competence

The perceived expertise of doctors and nurses was consistently contrasted with the limited training of TBAs and informal providers.

Chioma (32, university-educated) observed:

Doctors and nurses here are formally trained. Unlike local midwives and quack doctors, they are competent.

#### Positive past experiences

Women also highlighted how favourable past outcomes influenced their decision to return to facilities for subsequent pregnancies.

Mary (37, university-educated) explained:

Attending a healthcare facility for care… cannot be overemphasised. The presence of trained professionals gives a sense of security… Unlike home care and delivery, where the people involved lack clinical experience, a healthcare facility provides access to immediate medical interventions that could potentially save both the mother*'*s and the child*’*s life.

Collectively, these insights underscore the paradox at the heart of women's narratives: while mistreatment was common, the irreplaceable safety offered by skilled care compelled continued facility use.

### Barriers to maternal healthcare utilisation

Throughout the study, various obstacles to healthcare utilisation were identified by the participants, many of which were deeply rooted in systemic and societal issues. A significant number of these barriers were associated with negative attitudes from healthcare workers, the prevalence of domestic violence, and violations of patient confidentiality. These factors not only hindered women's ability to seek timely and adequate care but also contributed to feelings of mistrust and dissatisfaction with healthcare services. The finigs were reported under three sub-themes below:

#### Negative provider attitudes

Participants across age and educational groups consistently identified rudeness, neglect, and lack of empathy among healthcare workers as deterrents to facility-based care.

Amaka (29, a university student) reported:

*Healthcare workers are often rude and dismissive. It makes patients reluctant to return. A woman was publicly abused by a nurse. She was told that she was dirty and could not take care of her private area. This should not have been said in the hearing of other women, as it could discourage future use of the healthcare facility for antenatal, birth, and postpartum care.*

Ifeoma (30, university-educated) stated:

*As a patient in the hospital, even in an emergency situation, the healthcare staff expects you or your family to buy card and make some deposit before you can be attended to. This action by the health staff has left many women in need of prompt care death.*

Ada (34, secondary education) added:

*Healthcare staff in this country, particularly nurses, lack empathy and treat patients like rags. It's as if they forget that we are human beings, and we deserve care and respect just like anyone else. Their indifference makes the whole experience feel dehumanising and discourages many from seeking help when needed.*

#### Gender-based violence and domestic constraints

For some women, domestic violence intersected with inadequate healthcare response, further undermining trust in the system.

Justina (24) narrated:

*My husband caused three of the miscarriages I had. He always comes home drunk, and when I talk to him, he just starts pounding on me. I was rushed to this facility after noticing spotting following one of his attacks. However, when I was taken to the hospital by a good neighbour of mine, the healthcare professionals failed to attend to me immediately. As a result, I lost the baby and almost lost my life as well. The healthcare professionals see themselves as some kind of “gods” and are indifferent to their patients' concerns. I can't see myself coming back to this facility. It would be better if I patronised the chemist or relied on traditional birth attendants (TBAs) for their services.*

#### Confidentiality breaches and stigma

Three of the women noted that stigma surrounding reproductive health limited open discussion of sensitive issues. This silence reinforced under-utilisation, as women feared public shame or breaches of privacy.

Chioma (32) noted:

*I know I should be free to discuss reproductive health, but in reality, there is stigma everywhere. Even among educated people like the nurses, once your private issues become known, you are judged. That fear stops many of us from opening up.*

Justina (24) added:

*I wanted to tell the nurse about my bleeding, but I was afraid she would mention it in front of others. Once people hear such things, they look at you with shame, so I decided not to say anything.*

Mary (37) narrated:

*Some topics are too sensitive to raise, even with doctors. We fear that our issues will not remain confidential, and once it becomes public, the stigma is worse than the illness itself.*

One participant explained non-verbally through hesitation and silence that discussing issues such as miscarriage and intimate partner violence carried a social risk. Others echoed this sentiment, highlighting that breaches in confidentiality by healthcare workers amplified stigma.

#### Preference for alternative care

In response to mistreatment, women described shifting towards TBAs and private clinics, despite concerns over cost and clinical competence. TBAs, in particular, were valued for their cultural familiarity and perceived empathy.

Ijeoma (41, secondary education) stated:

*Sometimes doctors and nurses are just sitting around, refusing to attend to patients. It feels as if they are more interested in chatting amongst themselves than in providing the care we desperately need. This lack of urgency and professionalism only adds to the stress of being pregnant/sick and waiting for attention, making the whole experience even more frustrating and disheartening.*

Udoka (36, secondary education) added:

*There are a whole range of issues with the health system here, starting from supervision to overall management. Our health system still has a long way to go. In addition, there is the problem of doctors and nurses not prioritising the health of their patients. Many healthcare workers, especially nurses, seem more focused on making money from every situation than on the wellbeing of patients. Patients' welfare is often secondary to their personal interests.*

### Suggestions for improvement

In light of the challenges and barriers identified throughout the study, participants highlighted the need for comprehensive improvements in maternal healthcare services. They emphasised the importance of addressing both systemic issues within healthcare facilities and the interpersonal dynamics between healthcare providers and patients. Based on their experiences, the participants recommended several strategies to enhance the quality, accessibility, and overall patient experience of maternal healthcare services. This finings was repored uder three sub-themes.

#### Training on compassionate care

Participants stressed the importance of provider training in empathy and respectful maternity care.

Amaka (29) proposed:

Training and sensitisation for healthcare providers on compassionate care. Compassionate care motivates women to utilise health facilities.

#### Regular government monitoring

System-level oversight was identified as critical for upholding standards and ensuring the availability of essential equipment.

Justina (24) recommended:

*Frequent government-supported monitoring of healthcare facilities to uphold minimum standards and availability of necessary medical equipment.*

#### Policies upholding dignity, privacy, and respect

Ensuring women's rights to dignity and confidentiality was seen as essential for encouraging healthcare use.

Chioma (32) emphasised:

*Enforcing policies that guarantee patients' dignity, privacy, and respectful treatment.*

## Discussion

This study explored women's lived experiences of accessing maternal healthcare services in Nigeria, highlighting both facilitators and barriers within a complex socio-cultural and institutional context. The findings reveal a paradox: while women consistently acknowledged the irreplaceable role of skilled care in preventing maternal and neonatal deaths, their utilisation of such care was undermined by systemic inefficiencies, negative provider attitudes, breaches of confidentiality, and the persistence of stigma around sensitive reproductive health issues.

The recognition of skilled care as life-saving reflects women's deep awareness of the risks associated with childbirth and the limitations of home-based or unskilled delivery. Women's reliance on trained professionals and emergency interventions is consistent with evidence from sub-Saharan Africa, where the availability of skilled birth attendants significantly reduces maternal and neonatal mortality (13, 14). The finding aligns with the Theory of Planned Behaviour, as women's perceived control over health outcomes through access to skilled providers increased their intention to seek formal care (15). However, women's continued engagement with facilities despite poor experiences underscores that reliance on skilled care is often a matter of necessity rather than choice.

A critical barrier identified in this study was the widespread perception of disrespect and neglect by healthcare workers. The emotional and psychological toll of such treatment has been documented elsewhere as a deterrent to healthcare utilisation (19, 20). Breaches of confidentiality, alongside public shaming, intensified the stigma surrounding reproductive health matters, discouraging women from openly discussing sensitive issues such as miscarriage, bleeding, or intimate partner violence. This silence, as revealed in the results, perpetuates under-utilisation and echoes research from similar contexts where cultural taboos and fear of judgement constrained women's engagement with healthcare services (21, 22).

Domestic violence emerged as a compounding barrier, with some women experiencing pregnancy loss as a direct result of abuse. The failure of health workers to respond empathetically in these situations worsened women's trauma and eroded trust in the system. This intersection between gender-based violence and inadequate healthcare response highlights the need for a more integrated approach to maternal health, one that addresses not only clinical but also social determinants of health.

Women's recourse to TBAs and private providers, despite concerns over cost and clinical competence, reflects the inadequacies of government healthcare facilities. While TBAs are often problematised in policy discourse, participants in this study framed them as offering empathy, cultural familiarity, and respect, qualities they found lacking in formal facilities. Similar findings have been reported in Nigeria and other sub-Saharan African settings, where TBAs are valued for their accessibility and social embeddedness (14, 17, 23, 24). This underscores the importance of incorporating culturally sensitive perspectives into maternal health policy, recognising that women's choices are shaped not solely by medical safety but also by the interpersonal quality of care.

The findings highlight the urgent need for reforms that address both systemic and interpersonal dimensions of care. Training healthcare providers in respectful and compassionate care was a recurrent recommendation by participants and is supported by global evidence linking respectful maternity care with improved outcomes and greater patient satisfaction (25–27). At the systemic level, government-supported monitoring and enforcement of minimum standards are critical to ensure adequate staffing, equipment, and accountability. Importantly, policies must also enshrine dignity, privacy, and confidentiality, as these elements are central to building trust and overcoming the silences imposed by stigma.

## Strengths and limitations of the study

The study's qualitative design enabled a deep exploration of women's personal experiences with maternal healthcare, shedding light on sensitive issues often overlooked. The findings provide valuable insights into barriers and facilitators of healthcare utilisation, offering practical recommendations for improving services. It is particularly valuable for informing policy and healthcare reform in Nigeria.

The study also has some limitations. This study focused exclusively on women who were current users of formal healthcare facilities, which necessarily constrains the breadth of perspectives represented. The experiences of women who never access hospital-based maternal care, often due to financial barriers, geographic distance, cultural norms, or prior negative encounters, may diverge substantially from those reported here. Their voices remain absent from this analysis and constitute an important area for future inquiry.

Furthermore, because participants were recruited from a tertiary hospital, the findings should be interpreted with caution when applied to rural or primary-level contexts. While the thematic insights concerning healthcare worker attitudes, systemic inefficiencies, and socio-cultural dynamics may be transferable, they cannot be assumed to fully represent the experiences of non-users or women who rely primarily on traditional birth attendants. The purposive sampling of facility users may also introduce selection bias, as these women already demonstrated a degree of acceptance of biomedical care.

To extend the contribution of this study, future research should incorporate the perspectives of non-users, community stakeholders such as traditional birth attendants, and family members who often influence decision-making. Doing so would more fully reflect the principles of the Silences Framework by giving voice to those marginalised or excluded from mainstream healthcare discourse.

## Contribution and conclusion of the study

By exploring women's narratives across different demographic profiles, this study demonstrates that barriers and facilitators are not experienced uniformly. Younger, less-educated women were more vulnerable to stigma and public humiliation, while older, multiparous women often highlighted inefficiencies and neglect. This nuance adds depth to existing literature, which often treats women as a homogenous group, and strengthens calls for targeted, context-sensitive interventions.

This study reveals that women's utilisation of maternal healthcare in Nigeria is shaped by a tension between necessity and dissatisfaction: skilled care is recognised as essential, yet its promise is undermined by mistreatment, systemic failures, and cultural silences. Addressing these contradictions requires reforms that extend beyond infrastructure to encompass compassionate care, confidentiality, and cultural sensitivity. Such measures are essential for restoring trust in formal healthcare facilities and for ensuring that women not only access care but also experience it as respectful, safe, and empowering.

## Data Availability

The raw data supporting the conclusions of this article will be made available by the authors, without undue reservation.
